# Postoperative Adjuvant Chemoradiotherapy ± PD‐1 Inhibitor for Locally Advanced Head and Neck Squamous Cell Carcinoma

**DOI:** 10.1002/cnr2.70488

**Published:** 2026-02-27

**Authors:** Zhiqiang Wang, Qingqing He, Donghui Jiang, Sheng Cheng, Jingyu Gao, Yan Wang, Shuai Fu, Qing ying Cui, Yanli Yang, Li Lv, Yuchuan Xu, Yan Li, Rui Tian, Chunlei Ge, Rongqing Li

**Affiliations:** ^1^ Department of Radiotherapy Oncology First Affiliated Hospital of Kunming Medical University Kunming People's Republic of China; ^2^ Department of 2nd Otorhinolaryngology The First Affiliated Hospital of Kunming Medical University Kunming People's Republic of China; ^3^ Department of 1st Otorhinolaryngology First Affiliated Hospital of Kunming Medical University Kunming People's Republic of China; ^4^ Department of Oral and Maxillofacial Surgery Kunming Medical University School and Hospital of Stomatology Kunming China; ^5^ Department of Medical Oncology The Second Affiliated Hospital of Kunming Medical University Kunming Yunnan China; ^6^ Department of Cancer Biotherapy Center The Third Affiliated Hospital of Kunming Medical University (Tumor Hospital of Yunnan Province) Kunming People's Republic of China

**Keywords:** adjuvant therapy, head and neck squamous cell carcinoma, intensity‐modulated radiation therapy, PD‐1 Ab, postsurgical immunotherapy

## Abstract

**Background:**

Postsurgical immunotherapy for patients with locally advanced head and neck squamous cell carcinoma (HNSCC) remains elusive. This study assesses the value and significance of postoperative immunotherapy in the treatment of locally advanced HNSCC.

**Methods:**

In total, 212 patients with locally advanced HNSCC who underwent radical surgery were stratified into three treatment groups: adjuvant radiotherapy alone, adjuvant chemoradiotherapy (CRT), and adjuvant chemoradiotherapy plus immunotherapy (PD‐1 Ab). A comprehensive analysis was conducted to assess survival outcomes and prognostic factors across diverse patient cohorts.

**Results:**

Four patients were lost to follow‐up, with a follow‐up rate of 98.1% and a median follow‐up time of 41 months (IQR 24–68). A total of 64/212 individuals died, with cancer being the cause of 62 cases and noncancer causes accounting for the remaining two deaths; 62/212 (29.2%) patients experienced relapse and/or metastasis. The 3‐year OS rates for the radiotherapy group, CRT group, and CRT plus PD‐1 Ab group were 54.8%, 75.4%, and 82.2%, respectively. However, no statistically significant difference in OS or PFS was observed between the CRT and CRT + PD‐1 Ab groups (*p*_all_ > 0.05), although both were superior to radiotherapy alone (*p*_all_ < 0.05). Multivariate analysis indicated that age, smoking history, TNM stage and treatment method were independent prognostic factors for OS (*p*_all_ < 0.05). Smoking history and treatment methods were independent prognostic factors for PFS and DMFS (*p*_all_ < 0.05).

**Conclusion:**

PD‐1 Ab may contribute less to tumors with better treatment outcomes from concurrent chemoradiotherapy.

## 1 Research Background

In the early stages of the KEYNOTE‐012 [1] phase Ib and CheckMate‐141 [2] phase III clinical trials, immunotherapy significantly prolonged the OS of patients with recurrent/metastatic head and neck squamous cell carcinoma (HNSCC). The results of the KEYNOTE‐040 [3] and 048 [4] studies further confirmed the efficacy and safety of immune checkpoint inhibitors in the treatment of recurrent or metastatic HNSCC, and immune checkpoint inhibitors have been approved as the first‐line therapy for patients with recurrent or metastatic HNSCC. For patients with loco‐regionally advanced disease, radiotherapy is still the preferred treatment for head and neck tumors. The combination of immune checkpoint inhibitors and radiotherapy can have synergistic effects by promoting immune system recognition of tumor cells and reducing immunosuppression in the tumor microenvironment [5], thereby enhancing therapeutic efficacy. Based on the above‐mentioned studies, some scholars have proposed that immunotherapy will become an important breakthrough in improving the survival prognosis of patients with locally advanced HNSCC.

In a previous phase Ib study, pembrolizumab in combination with chemotherapy and radiotherapy was used to treat locally advanced HNSCC, which preliminarily confirmed the efficacy and safety of adjuvant chemoradiotherapy ± PD‐1 inhibitor in locally advanced patients [6] and merits further research and exploration. Draper et al. reported that compared with radiotherapy alone, immunotherapy combined with radiotherapy after surgery for patients with locally advanced HNSCC improved the 1‐year disease‐free survival of those in the intermediate‐risk group (96% vs. 69%) [7]. However, in the JAVELIN Head & Neck 100 [8], KEYNOTE‐412 [9], IMvoke 010 [10], and meta—analysis [11] studies, the combination of PD‐1/PD‐L1 Ab with standard chemoradiotherapy failed to significantly improve the efficacy of standard chemoradiotherapy in patients with locally advanced HNSCC. Through our analysis and comparison of the aforementioned studies, we have observed a notable distinction: in metastatic/recurrent disease, the systemic tumor burden is typically high—a context in which immune checkpoint inhibitors (ICIs) have demonstrated marked efficacy. In contrast, following curative‐intent concurrent chemoradiotherapy (CCRT), effective local control is usually achieved, and the remaining micrometastatic burden is likely to be low. Consequently, we speculate that this discrepancy may be that immune checkpoint inhibitors exert their primary effect by activating systemic anti‐tumor immunity to eradicate micrometastatic disease, whereas concurrent chemoradiotherapy is already highly effective in achieving locoregional control. Therefore, the incremental benefit of adding immunotherapy to robust local therapy might be more evident in preventing distant failure rather than further improving local control.

To test this hypothesis, we analyzed locally advanced HNSCC patients who underwent adjuvant chemoradiotherapy combined with immunotherapy following radical surgery. This study aimed to explore the clinical efficacy and identify prognostic factors influencing patient survival, thereby providing valuable insights for improving the treatment of locally advanced HNSCC.

## 2 Materials and Methods

### 2.1 Clinical Data

The Clinical Research Ethics Committee of the First Affiliated Hospital of Kunming Medical University approved this study (2024 Ethics Review L No. 76), and the participants provided written informed consent.

This is a single‐center, retrospective cohort study. In this study, we collected the clinical data of patients with locally advanced HNSCC who underwent radical surgery at our center between January 1, 2016, and July 31, 2023. Treatment allocation was not randomized. The postoperative adjuvant strategy (RT alone, CRT, or CRT + IO) for each patient was determined by a multidisciplinary tumor board based on a comprehensive assessment considering the national treatment guidelines prevailing at the time, the patient's performance status (ECOG), comorbidities, pathological risk features, and patient preference after detailed consultation regarding the benefits and risks of each option. The patients were divided into the adjuvant radiotherapy alone group (radiotherapy group), adjuvant chemoradiotherapy group (chemoradiotherapy group), and adjuvant chemoradiotherapy plus PD‐1 Ab group (chemoradiotherapy plus PD‐1 Ab group) according to the postoperative adjuvant strategies. The patient eligibility criteria were as follows: (1) had HNSCC confirmed by pathological examination; (2) had received surgical treatment with the goal of curative treatment prior to radiotherapy/chemoradiotherapy; (3) had a clinical stage of III/IVa, as defined by the American Joint Committee on Cancer Seventh Edition [12]; (4) had an ECOG PS of 0–2 points; and (5) were aged ≥ 18 years. The exclusion criteria were as follows: (1) patients who did not complete concurrent treatment as scheduled (not receiving radiotherapy for more than 1 week), irrespective of the number of chemotherapy or immunotherapy cycles; (2) patients with second primary malignant tumors; and (3) patients who had previously received immune checkpoint inhibitors.

### 2.2 Surgery

All patients underwent radical surgery with curative intent performed by Senior head and neck surgeons at our tertiary center. While surgical approaches varied by tumor site (e.g., laryngectomy, glossectomy, neck dissection), the principles of en bloc resection with clear margins were uniformly applied.

### 2.3 Radiotherapy

All patients were delivered using Elekta linear accelerators (intensity‐modulated radiation therapy, IMRT)with 6 MV X Ray. The target volume was delineated on the basis of the 2018 International Consensus on HNSCC [13], considering preoperative and postoperative CT/MRI or PET‐CT scans of the head and neck, as well as postoperative pathological results. Patients were immobilized using a thermoplastic head‐shoulder mask (Supine position with neck support). Daily cone‐beam CT (CBCT) was used for setup verification. The gross tumor volumes of the tumor bed (GTVtb) included positive surgical margins or macroscopic residual tumors after surgery. The high‐risk clinical target volume (CTV‐p1) was defined as the GTVtb plus a 5 mm margin. The low‐risk clinical target volume (CTV‐p2) was defined as the CTV‐p1 plus a 5‐mm margin. Clinical target volume n1 (CTV‐n1) was defined as the high‐risk infiltration lymphatic drainage area. Clinical target volume n2 (CTVn‐2) was defined as the low‐risk infiltration lymphatic drainage area. The target region of CTV‐n1 and CTV‐P1 were combined to form CTV1.

Uniform expansions of 0.3 cm from the GTV or CTVs were performed to create planning target volumes (PTVs). The prescribed doses were 66–70 Gy, 60–64 Gy, and 54–56 Gy in 30–33 fractions for PGTVtb, PCTV1, and PCTV2 derived from GTVtb, CTV1, and CTV2, respectively. Sequential integrated boost was used, with simultaneous delivery of different dose levels to PGTVtb, PCTV1, and PCTV2. All patients received one daily radiotherapy session, 5 days per week. Treatment planning was carried out using the Monaco (Elekta) systems by tumor radiotherapy physicists. Organ‐at‐risk (OAR) list and specific dose limits are shown in Table S9.

### 2.4 Systemic Therapy Drugs

During concurrent chemoradiotherapy, cisplatin was administered for two cycles. Dose specifications were as follows: cisplatin (100 mg/m^2^, Q3W) for chemotherapy alone and cisplatin (80 mg/m^2^, Q3W) for combined chemo‐immunotherapy. Chemotherapy could be postponed due to severe grade 3/4 treatment‐related toxicities. If the delay exceeded 2 weeks, chemotherapy was discontinued. In cases of intolerable toxicity, the dose could be reduced by one level (20%), with a maximum of two dose reductions permitted.

The immune‐checkpoint inhibitor selected is tislelizumab, administered at a dose of 200 mg every 3 weeks during concurrent radiotherapy and adjuvant maintenance, with the duration of maintenance therapy tailored to the patient's condition.

### 2.5 Statistical Analysis

Normally distributed data were analyzed using the Shapiro–Wilk test and are expressed herein as the mean ± standard deviation. Intergroup comparisons using one‐way analysis of variance (ANOVA) did not show a normal distribution. The median and interquartile range M (QL, QU) were calculated, and the Kruskal–Wallis H rank sum test was used to conduct intergroup comparisons. Count data are expressed as numbers (*n*) and rates (%), and intergroup comparisons were performed using the chi‐square test. Survival analysis was performed using the Kaplan–Meier method. The Cox proportional hazards regression model was used for multivariate survival analysis. All the statistical tests were bilateral, with a significance level of *p* < 0.05. Statistical analyses were performed using SPSS 26.0 software.

## 3 Results

### 3.1 Patient Characteristics

**FIGURE 1:**
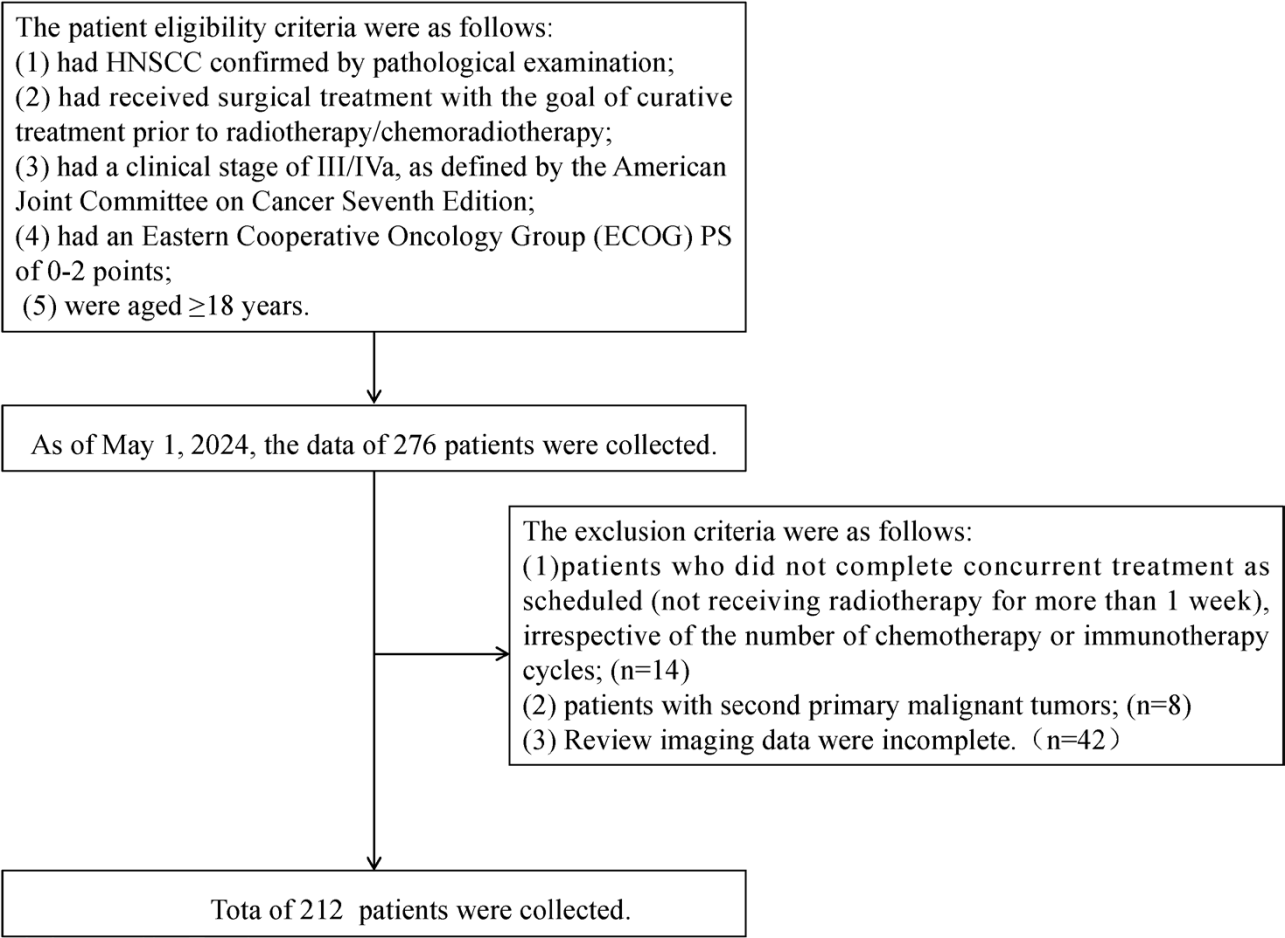
Research flowchart.

As of May 1, 2024, the data of 276 patients were collected. Of these, 64 patients met the exclusion criteria as follows: the review imaging data of 42 patients were incomplete, 14 patients failed to complete radiotherapy as planned, and another 8 patients had malignant thyroid tumors. After screening, a total of 212 patients were eligible for inclusion in the analysis (Figure 1). All patients were followed up through outpatient follow‐up records and telephone contact. Four patients were lost to follow‐up, with a follow‐up rate of 98.1% and a median follow‐up time of 41 months (IQR · 24–68). The male population constituted 86.7% (*n* = 184), whereas the female population accounted for 13.3% (*n* = 28). The male‐to‐female ratio was approximately 6:1. The median age of the patients was 57 years (range: 21–80 years). Among the patients, 92 (43.4%) were older than 60 years, while 120 (56.6%) were younger than 60 years. The distribution of pathogenesis locations was as follows: oral cancer in 58 (27.4%) patients, oropharyngeal cancer in 42 (19.8%), laryngeal cancer in 71 (33.4%), and hypopharyngeal carcinoma in 41 (19.4%). Stage III disease was observed in 110 (51.9%) patients, whereas stage IVa disease was present in 102 (48.1%) patients. The radiotherapy group consisted of 56 (26.4%) patients, the chemoradiotherapy group included 99 (46.7%) patients, and the chemoradiotherapy plus PD‐1 Ab group comprised 57 (26.9%) patients. No significant differences were found among the three groups regarding basic clinical information (*p*_all_ > 0.05). The median duration of adjuvant PD‐1 Ab was 6 months (interquartile range: 4–8 months), and the minimum and maximum treatment durations were 3 months and 38 months, respectively. Detailed information is provided in Table 1.

**TABLE 1:** Description and comparison of the baseline information of the patients.

Baseline information	Radiotherapy group (*n* = 56)	Chemoradiotherapy group (*n* = 99)	Combined immunotherapy group (*n* = 57)	χ^2^	*p*	Calibrated *p*‐value*
Age (years)				2.622	0.270	0.291
< 60	27 (48.2%)	61 (61.6%)	32 (56.1%)			
≥ 60	29 (51.8%)	38 (38.4%)	25 (43.9%)			
Sex				1.924	0.382	0.368
Male	46 (82.1%)	89 (89.9%)	49 (86.0%)			
Women	10 (17.9%)	10 (10.1%)	8 (14.0%)			
ECOG score				3.621	0.164	0.169
0–1	41 (73.2%)	73 (73.7%)	49 (86.0%)			
≥ 2	15 (26.8%)	26 (26.3%)	8 (14.0%)			
Smoking history				0.391	0.823	0.847
None	24 (42.9%)	42 (42.4%)	27 (47.4%)			
Yes	32 (57.1%)	57 (57.6%)	30 (52.6%)			
Alcohol consumption history				1.621	0.445	0.452
None	29 (51.8%)	61 (61.6%)	35 (61.4%)			
Yes	27 (48.2%)	38 (38.4%)	22 (38.6%)			
Delay in seeking medical attention				4.027	0.133	0.128
None	18 (32.1%)	46 (46.5%)	28 (49.1%)			
Yes	38 (67.9%)	53 (53.5%)	29 (50.9%)			
Comorbidities				4.842	0.089	0.087
None	35 (62.5%)	67 (67.7%)	46 (80.7%)			
Yes	21 (37.5%)	32 (32.3%)	11 (19.3%)			
Lymph node dissection				1.917	0.383	0.374
None	15 (26.8%)	35 (35.4%)	22 (38.6%)			
Yes	41 (73.2%)	64 (64.6%)	35 (61.4%)			
Tumor location				9.665	0.140	0.140
Larynx	24 (42.9%)	35 (35.4%)	12 (21.1%)			
Oral cavity	10 (17.9%)	27 (27.3%)	21 (36.8%)			
Oropharynx	13 (23.2%)	16 (16.2%)	13 (22.8%)			
Hypopharynx	9 (16.1%)	21 (21.2%)	11 (19.3%)			
Degree of differentiation				8.826	0.184	0.185
Well‐differentiated	17 (30.4%)	40 (40.4%)	27 (47.4%)			
Moderately differentiated	18 (32.1%)	37 (37.4%)	16 (28.1%)			
Poorly differentiated	16 (28.6%)	12 (12.1%)	9 (15.8%)			
Differentiation unclear	5 (8.9%)	10 (10.1%)	5 (8.8%)			
T stage				4.278	0.118	0.120
T_1_–T_2_	33 (58.9%)	43 (43.4%)	32 (56.1%)			
T_3_–T_4a_	23 (41.1%)	56 (56.6%)	25 (43.9%)			
N stage^a^				0.443	0.801	0.814
N_0_–N_1_	34 (60.7%)	59 (59.6%)	37 (64.9%)			
N_2_	22 (39.3%)	40 (40.4%)	20 (35.1%)			
TNM stage^b^				1.660	0.436	0.434
III	30 (53.6%)	47 (47.5%)	33 (57.9%)			
IVa	26 (46.4%)	52 (52.5%)	24 (42.1%)			

*Note:* a and b: Patients with stage N3 and IVb are not suitable for surgical treatment and are therefore excluded. Baseline characteristics were well balanced across the three groups (*p*_all_ > 0.05, Table 1), reducing concerns about major confounding by these known factors despite non‐random allocation.

* Bonferroni correction.

### 3.2 Patient Comorbidities

Among the 212 patients with locally advanced HNSCC, comorbidities were observed in 64 patients, with a majority (53.1%) having a history of high blood pressure (34 patients), followed by diabetes (18 patients, 28.1%). Ten patients (15.7%) had hypertension and diabetes, as well as cardiopulmonary diseases (2 patients, 3.1%), including coronary heart disease and chronic obstructive pulmonary disease. Stratification of the data by age group revealed that the proportion of comorbidities was slightly greater among patients aged 60 years and above, and hypertension remained the primary manifestation of comorbidities in both age groups (see Table 2).

**TABLE 2:** Comorbidities in different age groups.

Groups	Whole group of patients	< 60	≥ 60
Number of cases	64	27	37
High blood pressure	34 (53.1%)	15 (55.5%)	19 (51.3%)
Diabetes	18 (28.1%)	8 (29.6%)	10 (27.0%)
Cardiopulmonary disease	2 (3.1%)	1 (3.8%)	1 (2.7%)
Two comorbidities	10 (15.7%)	3 (11.1%)	7 (19.0%)

### 3.3 Survival

**FIGURE 2:**
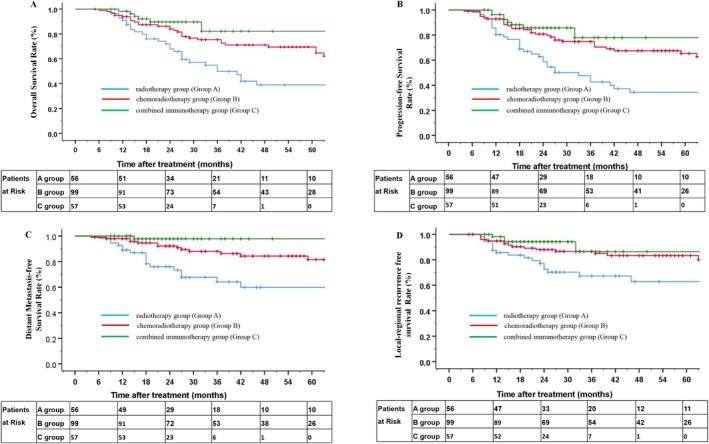
Kaplan–Meier curves for all enrolled patients. Kaplan–Meier curves of overall survival (A), progression‐free survival (B), distant metastasis‐free survival (C), and locoregional recurrence‐free survival (D) for patients with locally advanced HNSCC in the postoperative adjuvant radiotherapy group, chemoradiotherapy group, and chemoradiotherapy plus PD‐1 Ab group.

Among the 212 patients, a total of 64 individuals succumbed to their conditions, with cancer being the cause of death in 62 patients and noncancer causes accounting for the remaining two deaths (one due to COVID‐19 and one due to an unknown cause). However, no statistically significant difference in OS or PFS was observed between the chemoradiotherapy and chemoradiotherapy plus PD‐1 Ab groups (*p*_all_ > 0.05), although both were superior to radiotherapy alone (*p*_all_ < 0.05). In the radiotherapy group, chemoradiotherapy group, and CT plus PD‐1 Ab group, the 3‐year OS rates were 54.8%, 75.4%, and 82.2%, respectively. The 3‐year PFS rates for the three groups were 47.6%, 74.8%, and 77.9%; the 3‐year DMFS rates for the three groups were 64.1%, 88.0%, and 97.9%; and the 3‐year LRFS rates for the three groups were 67.3%, 86.6%, and 86.4%, respectively. Table 3 lists the details. In the chemoradiotherapy plus PD‐1 Ab group, the DMFS increased by 9.9% compared with the chemoradiotherapy group, indicating a potential benefit. The survival rates of patients in the three groups are shown in Table 4, and survival curves are shown in Figure 2.

**TABLE 3:** Survival rates among the three treatment modalities.

Parameters	Radiotherapy group	Chemoradiotherapy group	Chemoradiotherapy plus PD‐1 Ab group
1 year‐OS (%)	91.1%	93.9%	98.2%
2 year‐OS (%)	72.0%	86.3%	89.6%
3 year OS (%)	54.8%	75.4%	82.2%
1 year PFS (%)	85.7%	92.9%	96.4%
2 year PFS (%)	62.9%	80.8%	85.7%
3 year PFS (%)	47.6%	74.8%	77.9%
1 year DMFS (%)	92.8%	98.0%	100%
2 year DMFS (%)	76.0%	92.2%	97.9%
3 year DMFS (%)	64.1%	88.0%	97.9%
1 year LRFS (%)	87.5%	94.8%	98.2%
2 year LRFS (%)	77.1%	87.9%	94.3%
3 year LRFS (%)	67.3%	86.6%	86.4%

**TABLE 4:** Comparison of the survival rates among the three treatment modalities.

Parameters	*χ* ^2^	*p*
OS		
Radiotherapy vs. Chemoradiotherapy	8.553	0.003
Radiotherapy vs. Chemoradiotherapy plus PD‐1 Ab	9.215	0.002
Chemoradiotherapy vs. Chemoradiotherapy plus PD‐1 Ab	1.568	0.210
PFS		
Radiotherapy vs. Chemoradiotherapy	12.140	< 0.001
Radiotherapy vs. Chemoradiotherapy plus PD‐1 Ab	10.847	0.001
Chemoradiotherapy vs. Chemoradiotherapy plus PD‐1 Ab group	0.934	0.334
DMFS		
Radiotherapy vs. Chemoradiotherapy	9.159	0.002
Radiotherapy vs. Chemoradiotherapy plus PD‐1 Ab	12.628	< 0.001
Chemoradiotherapy vs. Chemoradiotherapy plus PD‐1 Ab	2.665	0.103
LRFS		
Radiotherapy vs. Chemoradiotherapy	5.424	0.020
Radiotherapy vs. Chemoradiotherapy plus PD‐1 Ab	6.240	0.012
Chemoradiotherapy vs. Chemoradiotherapy plus PD‐1 Ab	0.741	0.389

### 3.4 Relapse and Metastasis

As of the follow‐up date, 62 patients had developed disease progression. The recurrence and/or metastasis rate was 29.2% (62/212). In the radiotherapy group, 31/56 patients experienced recurrence and/or metastasis (55.3%). There were 14 patients with local recurrence (45.1%), 14 patients with distant metastasis (45.1%), 3 patients with recurrence and metastasis (9.8%), 14 patients who died of recurrence (45.1%), and 10 patients who died of distant metastasis (32.2%). Three patients (9.8%) died of recurrence combined with distant metastasis; among the 31 patients with recurrence and metastasis, 13 had stage III disease, and 18 had stage IVa disease.

In the chemoradiotherapy group, 26/99 patients experienced recurrence and/or metastasis (26.2%). Thirteen patients experienced local recurrence (50.0%), 11 patients experienced distant metastasis (42.3%), 2 patients experienced recurrence and metastasis (7.7%), 12 patients died of recurrence (46.1%), and 10 patients (38.4%) died of recurrence and distant metastasis (7.7%). Among 26 patients with recurrence and metastasis, 4 had stage III disease, and 22 had stage IVa disease.

In the chemoradiotherapy plus PD‐1 Ab group, 5/57 patients experienced recurrence and/or metastasis (8.7%). Four patients experienced local recurrence (80.0%), 1 patient experienced distant metastasis (20.0%), and 4 patients died of recurrence (80.0%). Among the 4 patients who experienced recurrence, 2 patients had stage III disease, and 3 patients had stage IVa disease (see Table 5).

**TABLE 5:** Relapse patterns according to the treatment modality (%).

Group	Radiotherapy group (*n* = 39)	Chemoradiotherapy group (*n* = 35)	Chemoradiotherapy plus PD‐1 Ab group (*n* = 7)
Local recurrence	17 (43.5%)	20 (57.2%)	5 (71.4%)
Distant metastasis	16 (41.1%)	13 (37.1%)	2 (28.5%)
Local + distant metastasis	6 (15.4%)	2 (5.7%)	0 (0%)

### 3.5 Univariate and Multivariate Survival Analysis

The OS, PFS, DMFS, and LRFS of the 212 patients were analyzed by univariate and multivariate Cox regression models. The survival curves of the patients are shown in Figure S1. A log‐rank univariate survival analysis was performed using OS and PFS as the observation endpoints. Age, ECOG score, smoking history, alcohol consumption history, neck lymph node dissection, T stage, N stage, TNM stage, and treatment were poor prognostic factors for OS in patients with locally advanced HNSCC (*p*_all_ < 0.05). Additionally, age, ECOG score, comorbidities, smoking history, alcohol consumption history, neck lymph node dissection, N stage, TNM staging, and treatment methods were poor prognostic factors for PFS in patients with locally advanced HNSCC (*p*_all_ < 0.05) (Table S1, Table S2). The results of the log‐rank univariate survival analysis using DMFS and LRFS as the observation endpoints are shown in Tables S3 and S4.

The detailed results of the multivariate Cox proportional hazards analyses, identifying independent prognostic factors for OS, PFS, DMFS, and LRFS, are presented in Tables S5–S8. Multivariate analysis indicated that age, smoking history, TNM stage, and treatment method were independent prognostic factors for OS (*p*_all_ < 0.05). Smoking history and treatment methods were found to be independent prognostic factors for PFS (*p*_all_ < 0.05). The results of the multivariate analysis of DMFS and LRFS are shown in Tables S7 and S8.

## 4 Discussion

Postoperative adjuvant radiotherapy or combination chemotherapy for patients with locally advanced HNSCC is currently the main treatment approach. However, the potential benefits of postoperative adjuvant immunotherapy in these patients remain unclear. Although this was an evidence level B study, our findings suggested that the benefit of PD‐1 inhibitors in this setting may be primarily attributable to enhanced systemic disease control, while the contribution to locoregional control might have been largely provided by the concurrent chemoradiotherapy itself.

We observed that the OS of patients with locally advanced HNSC who received postoperative radiotherapy alone was poor. The addition of chemotherapy significantly improved the local recurrence and metastasis of the lesion in patients with locally advanced disease. This finding is consistent with the results of the RTOG 9501 clinical trial [14] and the EORTC 22931 trial [15]. However, adding immunotherapy to postoperative adjuvant chemoradiotherapy did not result in improved survival. This result is approximately the same as the recent IMvoke 010 [10] trial. In addition, in the JAVELIN Head and Neck‐100 prospective clinical trial [8] and the KEYNOTE‐412 clinical study [16] adjuvant chemoradiotherapy ± PD‐1/PD‐L1 Ab did not prolong PFS or OS in patients with unresectable locally advanced HNSCC. The collective negative results of these trials, which administered immunotherapy concurrently with or as maintenance after definitive chemoradiotherapy, suggest that simply adding immune checkpoint inhibition to or after an already potent local treatment regimen may provide limited incremental survival benefit for an unselected population. This aligns with our hypothesis that the therapeutic window for immunotherapy's added value might be narrower in contexts where chemoradiotherapy itself provides robust disease control.

However, there was a potential benefit observed in the DMFS. In our study, the 3‐year DMFS of the chemoradiotherapy group was 88.0%, whereas it was 97.9% for the chemoradiotherapy plus PD‐1 Ab group (*p* = 0.103), suggesting a potential survival benefit of immunotherapy on DMFS. On the other hand, studies such as KEYNOTE‐040 [3] and 048 [4] mainly focused on recurrent and metastatic HNSCC, where more than 70% of patients had distant metastases and none received concurrent chemoradiotherapy. This may be one of the main reasons why immunotherapy has shown benefits in recurrent or metastatic HNSCC patients. These findings further support our hypothesis that immunotherapy may contribute less to tumors with better treatment outcomes from concurrent chemoradiotherapy. Although positive treatment outcomes have been observed in other tumor types (such as nasopharyngeal cancer) with high cure rates, the control group data [17] in these studies were comparatively lower than the previously reported data [18] from patients receiving equivalent treatments.

The recent positive result of the NIVOPOSTOP trial, demonstrating a disease‐free survival benefit with nivolumab added to postoperative chemoradiotherapy, provides a crucial counterpoint and helps refine the interpretation of our findings [19]. A key distinction lies in patient selection: NIVOPOSTOP exclusively enrolled patients with high‐risk pathological features (e.g., extranodal extension, positive margins), a highly concentrated risk population. In contrast, our cohort included the broader spectrum of stage III/IVa disease. This suggests that the benefit of adjuvant immunotherapy may be most pronounced in a defined, very high‐risk subgroup, potentially explaining the more modest overall effect observed in our heterogeneous population. Furthermore, variations in real‐world adherence and integration intensity compared to a clinical trial setting may impact survival outcomes. Therefore, our results do not contradict the efficacy of immunotherapy in the adjuvant setting but rather underscore that its measurable impact on survival endpoints like OS and PFS may be contingent upon rigorous patient risk stratification and the depth of therapeutic integration.

In addition, the multivariate analysis identified several independent prognostic factors, the recognition of which carries direct clinical implications. This study revealed a poorer prognosis in elderly patients who had a smoking history and more advanced disease. Patients who received adjuvant chemoradiotherapy ± PD‐1 inhibitor had a longer OS than patients who received radiotherapy alone. Elderly patients with HNSCC often have comorbidities, which can lead to complications during treatment and make it difficult for these individuals to complete treatment. In the present study, 64 (30.2%) patients had comorbidities, 37 of whom were ≥ 60 years old, and these patients mainly had hypertension and diabetes. A survey on the association between comorbidities and the prognosis of 12 623 patients with HNSCC revealed that 36% of patients had comorbidities, and the 5‐year survival rate of patients with comorbidities was only 33%. The survival rate significantly decreased with increasing levels of comorbidities (*p* < 0.001) [20]. Comorbidities may affect the treatment choices of patients, especially older patients who are more inclined toward conservative or palliative treatments due to their comorbid conditions. This is an important reason for reduced treatment intensity among these patients [21]. According to a large population analysis involving 1287 patients, comorbidities were more common in those who were between 60 and 70 years of age. Hypertension was the most common comorbidity, while one‐fourth of the patients had chronic obstructive pulmonary disease; both diseases significantly affected patient survival time (*p* < 0.0001) [22]. Tseng and Sobti et al. [23, 24] also reported that patients with diabetes tend to have a worse prognosis than those without diabetes. This study also revealed lower survival rates in patients with comorbidities and multiple complications, especially elderly patients who had preexisting comorbidities before treatment. Therefore, it is crucial to assess the presence and severity of comorbidities when caring for and treating patients with newly diagnosed locally advanced HNSCC.

The findings of this study should be interpreted considering its inherent limitations. Firstly, this was a retrospective, single‐center analysis, which is susceptible to selection bias. Secondly, key biomarkers, such as tumor PD‐L1 expression and HPV status—were not available for analysis, precluding an evaluation of their potential predictive or prognostic role in this adjuvant setting. Thirdly, the study was not specifically powered to conduct robust subgroup analyses to identify which patients might derive the greatest benefit from combined modality therapy. These factors may limit the generalizability of our conclusions and highlight the need for validation in prospective, ideally biomarker‐integrated, clinical trials.

In summary, despite these limitations, our real‐world data suggested that the efficacy of PD‐1 inhibitors may be mainly attributable to systemic treatment, while the local treatment effect might be covered by concurrent chemoradiotherapy, particularly in tumors exhibiting sensitivity to chemoradiotherapy. This hypothesis‐generating finding warrants further investigation in prospective studies to refine patient selection for this intensive multimodal approach.

## Author Contributions

**Zhiqiang Wang:** conception and design, methodology, software, investigation, project administration, writing – review and editing, writing – original draft, funding acquisition. **Qingqing He:** collection and assembly of data, software, investigation, project administration, writing – original draft. **Donghui Jiang:** writing – original draft, collection and assembly of data, software, investigation, project administration. **Sheng Cheng:** writing – original draft, collection and assembly of data, investigation, validation, formal analysis. **Jingyu Gao:** collection and assembly of data, investigation, validation, formal analysis. **Yan Wang:** collection and assembly of data, investigation, validation, formal analysis. **Shuai Fu:** collection and assembly of data, investigation, validation, formal analysis. **Qing ying Cui:** collection and assembly of data, investigation, validation, formal analysis. **Yanli Yang:** investigation, collection and assembly of data. **Li Lv:** investigation, collection and assembly of data. **Yuchuan Xu:** collection and assembly of data. **Yang Li:** collection and assembly of data. **Rui Tian:** collection and assembly of data. **Chunlei Ge:** methodology, software, project administration, writing – review and editing, writing – original draft, quality control of data, funding acquisition. **Rongqing Li:** methodology, software, project administration, writing – review and editing, quality control of data, funding acquisition.

## Funding

The present study was partially supported by the 535 Talent Project of First Affiliated Hospital of Kunming Medical University (2023535Q05), the Yunnan Fundamental Research Projects (grant no. 202301AT070130), the Yunnan Fundamental Research Kunming Medical University Projects (grant no. 202301AY070001‐115), the Doctoral Research Start‐up Fund for First Affiliated Hospital of Kunming Medical University (grant no. 2021BS005), the National Natural Science Foundation of China (grant nos. 82260462, 82460606), the Yunnan Health Training Project of High Level Talents (H‐202423), and the Precision Radiotherapy Science and Technology Innovation Team of Yunnan Universities (grant no. K12322113).

## Ethics Statement

The Clinical Research Ethics Committee of the First Affiliated Hospital of Kunming Medical University approved this study (2024 Ethics Review L No. 76). This research is conducted in compliance with the Declaration of Helsinki.

## Consent

The participants provided written informed consent.

## Conflicts of Interest

The authors declare no conflicts of interest.

## Supporting information


**Figure S1:** Kaplan–Meier curves of overall survival (A), progression‐free survival (B), distant metastasis‐free survival (C), and locoregional recurrence‐free survival (D) for all patients with locally advanced HNSCC.

**Table S1:** Effect of hormones on OS in 212 patients with locally advanced HNSCC.
**Table S2:** Effect of clinical factors on PFS in 212 patients with locally advanced HNSCC.
**Table S3:** Relationships between clinical factors and DMFS in 212 patients with locally advanced HNSCC.
**Table S4:** Relationships between clinical factors and LRFS in 212 patients with locally advanced HNSCC.
**Table S5:** Prognosis of 212 patients with locally advanced HNSCC according to multivariate analysis (OS).
**Table S6:** Prognostic multivariate analysis (PFS) of 212 patients with locally advanced HNSCC.
**Table S7:** Prognosis of 212 patients with locally advanced HNSCC according to multivariate analysis (DMFS).
**Table S8:** Prognosis of 212 patients with locally advanced HNSCC according to multivariate analysis (LRFS).
**Table S9:** Normal tissue dose constraints by structure.

## Data Availability

The data that supports the findings of this study are available in the Supporting Information of this article.
