# Supplementary material for: Postoperative Adjuvant Chemoradiotherapy ± PD‐1 Inhibitor for Locally Advanced Head and Neck Squamous Cell Carcinoma
Source: Cancer Rep (Hoboken). 2026 Feb 27;9(3):e70488. doi: 10.1002/cnr2.70488 (PMC12947766; doi:10.1002/cnr2.70488)
Supplement: Supplementary file 2 — Table S1: Effect of hormones on OS in 212 patients with locally advanced HNSCC. Table S2: Effect of clinical factors on PFS in 212 patients with locally advanced HNSCC. Table S3: Relationships between clinical factors and DMFS in 212 patients with locally advanced HNSCC. Table S4: Relationships between clinical factors and LRFS in 212 patients with locally advanced HNSCC. Table S5: Prognosis of 212 patients with locally advanced HNSCC according to multivariate analysis (OS). Table S6: Prognostic multivariate analysis (PFS) of 212 patients with locally advanced HNSCC. Table S7: Prognosis of 212 patients with locally advanced HNSCC according to multivariate analysis (DMFS). Table S8: Prognosis of 212 patients with locally advanced HNSCC according to multivariate analysis (LRFS). Table S9: Normal tissue dose constraints by structure. [file CNR2-9-e70488-s002.docx]

**Table S1. Effect of hormones on OS in 212 patients with locally advanced HNSCC**

| **Group** | **n** | **1-OS** | **2-OS** | **3-OS** | **𝜒^2^** | ***P*** |
| --- | --- | --- | --- | --- | --- | --- |
| **Age (years)** |  |  |  |  | 15.115 | ＜0.001 |
| ＜60 | 120 | 96.7% | 87.9% | 78.0% |  |  |
| ≥60 | 92 | 91.1% | 75.6% | 59.5% |  |  |
| **ECOG score** |  |  |  |  | 22.631 | ＜0.001 |
| 0-1 | 163 | 95.0% | 85.7% | 75.3% |  |  |
| ≥2 | 49 | 91.6% | 69.0% | 48.5% |  |  |
| **Smoking history** |  |  |  |  | 22.217 | ＜0.001 |
| None | 93 | 97.8% | 91.5% | 82.5% |  |  |
| Yes | 119 | 91.5% | 74.6% | 60.0% |  |  |
| **Alcohol consumption history** |  |  |  |  | 19.427 | ＜0.001 |
| None | 125 | 96.8% | 91.1% | 77.5% |  |  |
| Yes | 87 | 90.6% | 68.7% | 57.3% |  |  |
| **Lymph node dissection** |  |  |  |  | 8.791 | 0.003 |
| None | 140 | 94.2% | 77.7% | 63.4% |  |  |
| Yes | 72 | 97.2% | 86.7% | 81.7% |  |  |
| **T stage** |  |  |  |  | 1.020 | 0.312 |
| T_1-2_ | 108 | 96.3% | 84.4% | 75.5% |  |  |
| T_3-4_ | 104 | 94.2& | 80.6% | 62.4% |  |  |
| **N stage** |  |  |  |  | 35.823 | ＜0.001 |
| N_0_-N_1_ | 130 | 97.6% | 90.4% | 81.5% |  |  |
| N_2_ | 82 | 91.4& | 70.5% | 51.8% |  |  |
| **TNM stage** |  |  |  |  | 35.293 | ＜0.001 |
| III | 110 | 99.1% | 93.8% | 83.7% |  |  |
| IVa | 102 | 90.1% | 72.0% | 54.8% |  |  |
| **Treatment methods** |  |  |  |  | 13.905 | 0.001 |
| Radiotherapy | 56 | 91.1% | 72.0% | 54.8% |  |  |
| Chemoradiotherapy | 99 | 93.9% | 86.3% | 75.4% |  |  |
| Chemoradiotherapy plus PD-1 Ab | 57 | 98.2% | 89.6% | 82.2% |  |  |

**Table S2. Effect of clinical factors on PFS in 212 patients with locally advanced HNSCC**

| **Group** | **n** | **1-PFS** | **2-PFS** | **3-PFS** | **𝜒^2^** | ***P*** |
| --- | --- | --- | --- | --- | --- | --- |
| **Age (years)** |  |  |  |  | 11.068 | 0.001 |
| ＜60 | 120 | 94.1% | 82.7% | 73.9% |  |  |
| ≥60 | 92 | 87.8% | 69.4% | 58.6% |  |  |
| **ECOG score** |  |  |  |  | 29.581 | ＜0.001 |
| 0-1 | 163 | 92.6% | 83.3% | 73.9% |  |  |
| ≥2 | 49 | 83.3% | 52.8% | 40.2% |  |  |
| **Smoking history** |  |  |  |  | 24.937 | ＜0.001 |
| None | 93 | 96.7% | 88.3% | 82.2% |  |  |
| Yes | 119 | 88.1% | 68.0% | 54.7% |  |  |
| **Alcohol consumption history** |  |  |  |  | 22.736 | ＜0.001 |
| None | 125 | 95.2% | 86.1% | 76.9% |  |  |
| Yes | 87 | 87.2% | 63.7% | 50.1% |  |  |
| **Comorbidities** |  |  |  |  | 4.624 | 0.032 |
| None | 148 | 91.8% | 80.8% | 72.3% |  |  |
| Yes | 64 | 90.5% | 66.1% | 52.7% |  |  |
| **Lymph node dissection** |  |  |  |  | 7.485 | 0.006 |
| Yes | 72 | 91.5% | 84.5% | 79.6% |  |  |
| None | 140 | 89.9% | 73.3% | 59.1% |  |  |
| **T stage** |  |  |  |  | 1.566 | 0.211 |
| T_1-2_ | 108 | 92.5% | 75.9% | 73.0% |  |  |
| T_3-4_ | 104 | 90.3& | 71.5% | 59.1% |  |  |
| **N stage** |  |  |  |  | 29.155 | ＜0.001 |
| N_0_-N_1_ | 130 | 95.3% | 85.3% | 77.5% |  |  |
| N_2_ | 82 | 85.7% | 62.4% | 50.7% |  |  |
| **TNM stage** |  |  |  |  | 29.769 | ＜0.001 |
| III | 110 | 98.2% | 88.7% | 79.9% |  |  |
| IVa | 102 | 84.1% | 64.5% | 53.1% |  |  |
| **Treatment methods** |  |  |  |  | 17.746 | ＜0.001 |
| Radiotherapy | 56 | 85.7% | 62.9% | 47.6% |  |  |
| Chemoradiotherapy | 99 | 92.9% | 80.8% | 74.8% |  |  |
| Chemoradiotherapy plus PD-1 Ab | 57 | 96.4% | 85.7% | 77.9% |  |  |

**Table S3. Relationships between clinical factors and DMFS in 212 patients with locally advanced HNSCC**

| **Group** | **n** | **1-DMFS** | **2-DMFS** | **3-DMFS** | **𝜒^2^** | ***P*** |
| --- | --- | --- | --- | --- | --- | --- |
| **Age (years)** |  |  |  |  | 5.264 | 0.022 |
| ＜60 | 120 | 97.5% | 93.5% | 87.7% |  |  |
| ≥60 | 92 | 94.4% | 83.4% | 77.6% |  |  |
| **ECOG score** |  |  |  |  | 22.498 | ＜0.001 |
| 0-1 | 163 | 98.1% | 95.1% | 88.8% |  |  |
| ≥2 | 49 | 89.5% | 68.3% | 64.5% |  |  |
| **Smoking history** |  |  |  |  | 16.871 | ＜0.001 |
| None | 93 | 98.8% | 95.2% | 93.9% |  |  |
| Yes | 119 | 94.9% | 85.4% | 74.8% |  |  |
| **Alcohol consumption history** |  |  |  |  | 14.270 | ＜0.001 |
| None | 125 | 99.2% | 94.7% | 89.8% |  |  |
| Yes | 87 | 91.7% | 80.5% | 71.7% |  |  |
| **Lymph node dissection** |  |  |  |  | 5.382 | 0.020 |
| Yes | 72 | 98.5% | 96.6% | 91.0% |  |  |
| None | 140 | 94.9% | 85.4% | 79.4% |  |  |
| **T stage** |  |  |  |  | 0.728 | 0.393 |
| T_1-2_ | 108 | 97.2% | 88.5% | 87.0% |  |  |
| T_3-4_ | 104 | 97.1& | 88.4% | 79.1% |  |  |
| **N stage** |  |  |  |  | 8.899 | 0.003 |
| N_0_-N_1_ | 130 | 99.2% | 94.8% | 88.6% |  |  |
| N_2_ | 82 | 91.2% | 79.6% | 74.3% |  |  |
| **TNM stage** |  |  |  |  | 9.078 | 0.003 |
| III | 110 | 99.1% | 95.0% | 89.6% |  |  |
| IVa | 102 | 92.9% | 82.2% | 75.4% |  |  |
| **Treatment methods** |  |  |  |  | 17.404 | ＜0.001 |
| Radiotherapy | 56 | 92.8% | 76.0% | 64.1% |  |  |
| Chemoradiotherapy | 99 | 98.0% | 92.2% | 88.0% |  |  |
| Chemoradiotherapy plus PD-1 Ab | 57 | 100% | 97.9% | 97.9% |  |  |

**Table S4. Relationships between clinical factors and LRFS in 212 patients with locally advanced HNSCC**

| **Group** | **n** | **1-LRFS** | **2-LRFS** | **3-LRFS** | **𝜒^2^** | ***P*** |  |
| --- | --- | --- | --- | --- | --- | --- | --- |
| **Age (years)** |  |  |  |  | 4.173 | 0.041 | |
| ＜60 | 120 | 96.6% | 88.8% | 84.8% |  |  | |
| ≥60 | 92 | 88.9% | 83.4% | 78.3% |  |  | |
| **ECOG score** |  |  |  |  | 12.189 | ＜0.001 |  |
| 0-1 | 163 | 95.0% | 89.8% | 85.8% |  |  |  |
| ≥2 | 49 | 87.5% | 71.8% | 68.4% |  |  |  |
| **Smoking history** |  |  |  |  | 9.327 | 0.002 |  |
| None | 93 | 96.7% | 94.3% | 89.3% |  |  |  |
| Yes | 119 | 90.5% | 81.2% | 73.7% |  |  |  |
| **Alcohol consumption history** |  |  |  |  | 8.379 | 0.004 | |
| None | 125 | 95.9% | 91.2% | 88.7% |  |  | |
| Yes | 87 | 90.6% | 79.4% | 70.7% |  |  | |
| **T stage** |  |  |  |  | 0.437 | 0.509 | |
| T_1-2_ | 108 | 94.3% | 87.8% | 83.6% |  |  | |
| T_3-4_ | 104 | 92.2& | 83.5% | 80.0% |  |  | |
| **N stage** |  |  |  |  | 23.466 | ＜0.001 |  |
| N_0_-N_1_ | 130 | 96.9% | 93.0% | 88.9% |  |  |  |
| N_2_ | 82 | 88.7% | 76.1% | 66.5% |  |  |  |
| **TNM stage** |  |  |  |  | 21.567 | ＜0.001 |  |
| III | 110 | 99.1% | 93.5% | 90.1% |  |  |  |
| IVa | 102 | 86.8% | 77.5% | 69.8% |  |  |  |
| **Treatment methods** |  |  |  |  | 9.136 | 0.010 |  |
| Radiotherapy | 56 | 87.5% | 77.1% | 67.3% |  |  |  |
| Chemoradiotherapy | 99 | 94.8% | 87.9% | 86.6% |  |  |  |
| Chemoradiotherapy plus PD-1 Ab | 57 | 98.2% | 94.3% | 86.4% |  |  |  |

**Table S5. Prognosis of 212 patients with locally advanced HNSCC according to multivariate analysis (OS)**

| **Variable** | **Regression coefficient** | **HR** | **95%CI**  **Lower limit Upper limit** | ***P* value** |
| --- | --- | --- | --- | --- |
| **Age** (＜60 vs. ≥60) | 0.916 | 2.475 | 1.459 4.280 | 0.001 |
| **N stage** (N_0-1_ vs. N_2_) | 0.535 | 1.766 | 0.687 4.242 | 0.249 |
| **TNM stage** (Ⅲ vs. IVa) | 1.029 | 2.755 | 1.051 7.456 | 0.039 |
| **Cervical lymph node dissection** (no vs. yes) | -0.181 | 0.846 | 0.413 1.688 | 0.615 |
| **Smoking** (no vs. yes) | 1.061 | 3.073 | 1.373 6.064 | 0.005 |
| **Alcohol consumption (no vs. yes)** | 0.193 | 1.097 | 0.637 2.311 | 0.557 |
| **ECOG score** (0-1 vs. ≥2) | 0.007 | 1.018 | 0.559 1.815 | 0.981 |
| **Treatment methods** (Radiotherapy vs. Chemoradiotherapy) | -0.770 | 0.458 | 0.266 0.807 | 0.007 |
| **Treatment methods** (Radiotherapy vs. Chemoradiotherapy plus PD-1 Ab) | -1.067 | 0.343 | 0.137 0.863 | 0.023 |

**Table S6. Prognostic multivariate analysis (PFS) of 212 patients with locally advanced HNSCC**

| **Variable** | **Regression coefficient** | **HR** | **95%CI**  **Lower limit Upper limit** | ***P* value** |
| --- | --- | --- | --- | --- |
| **Age** (＜60 vs. ≥60) | 0.493 | 1.637 | 0.978 2.739 | 0.061 |
| **N stage** (N_0-1_ vs. N_2_) | 0.366 | 1.441 | 0.627 3.311 | 0.389 |
| **TNM stage** (Ⅲ vs. IVa) | 0.899 | 2.458 | 1.000 6.038 | 0.050 |
| **Cervical lymph node dissection** (no vs. yes) | -0.061 | 0.941 | 0.497 1.782 | 0.851 |
| **Smoking** (no vs. yes) | 0.242 | 1.274 | 0.763 2.128 | 0.354 |
| **Alcohol consumption (no vs. yes)** | 0.996 | 2.707 | 1.339 5.471 | 0.006 |
| **ECOG score** (0-1 vs. ≥2) | 0.278 | 1.321 | 0.723 2.415 | 0.366 |
| **Treatment methods** (Radiotherapy vs. Chemoradiotherapy) | 0.317 | 1.373 | 0.774 2.434 | 0.279 |
| **Treatment methods** (Radiotherapy vs. Chemoradiotherapy plus PD-1 Ab) | -0.926 | 0.396 | 0.234 0.670 | 0.001 |
| **TNM stage** (Ⅲ vs. IVa) | -1.038 | 0.354 | 0.158 0.795 | 0.012 |

**Table S7. Prognosis of 212 patients with locally advanced HNSCC according to multivariate analysis (DMFS)**

| **Variable** | **Regression coefficient** | **HR** | **95%CI**  **Lower limit Upper limit** | ***P* value** |
| --- | --- | --- | --- | --- |
| **Age** (＜60 vs. ≥60) | 0.458 | 1.581 | 0.713 3.505 | 0.260 |
| **N stage** (N_0-1_ vs. N_2_) | 0.355 | 1.426 | 0.402 5.064 | 0.583 |
| **TNM stage** (Ⅲ vs. IVa) | 0.552 | 1.737 | 0.452 6.682 | 0.422 |
| **Cervical lymph node dissection** (no vs. yes) | -0.191 | 0.826 | 0.288 2.369 | 0.723 |
| **Smoking** (no vs. yes) | 1.330 | 3.783 | 1.215 11.77 | 0.022 |
| **Alcohol consumption (no vs. yes)** | 0.343 | 1.409 | 0.581 3.417 | 0.448 |
| **ECOG score** (0-1 vs. ≥2) | 0.559 | 1.748 | 0.748 4.084 | 0.197 |
| **Treatment methods** (Radiotherapy vs. Chemoradiotherapy) | -1.112 | 0.329 | 0.152 0.711 | 0.005 |
| **Treatment methods** (Radiotherapy vs. Chemoradiotherapy plus PD-1 Ab) | -2.374 | 0.093 | 0.012 0.723 | 0.023 |

**Table S8. Prognosis of 212 patients with locally advanced HNSCC according to multivariate analysis (LRFS)**

| **Variable** | **Regression coefficient** | **HR** | **95%CI**  **Lower limit Upper limit** | ***P* value** |
| --- | --- | --- | --- | --- |
| **Age** (＜60 vs. ≥60) | 0.539 | 1.715 | 0.857 3.430 | 0.128 |
| **N stage** (N_0-1_ vs. N_2_) | 0.762 | 2.143 | 0.623 7.367 | 0.226 |
| **TNM stage** (Ⅲ vs. IVa) | 0.977 | 2.657 | 0.675 10.45 | 0.162 |
| **Smoking** (no vs. yes) | 0.906 | 2.474 | 0.963 6.355 | 0.060 |
| **Alcohol consumption (no vs. yes)** | 0.075 | 1.077 | 0.467 2.484 | 0.861 |
| **ECOG score** (0-1 vs. ≥2) | 0.230 | 1.259 | 0.595 2.663 | 0.547 |
| **Treatment methods** (Radiotherapy vs. Chemoradiotherapy) | -0.864 | 0.422 | 0.204 0.873 | 0.020 |
| **Treatment methods** (Radiotherapy vs. Chemoradiotherapy plus PD-1 Ab) | -1.172 | 0.310 | 0.101 0.951 | 0.041 |

**Table S9. Normal tissue dose constraints by structure.**

| **Structure** | **Dose constraints** | **Structure** | **Dose constraints** |
| --- | --- | --- | --- |
| Spinal cord | Dmax**^*^** ≤ 45 Gy | Temporomandibular Joint | Dmax < 70 Gy |
| Spinal cord_PRV | D1cc**†** ≤ 50 Gy | Parotid | Dmean < 26 Gy |
| Brain stem | Dmax ≤ 54 Gy | Parotid | V30**§** < 50% |
| Brain stem_PRV | D1cc ≤ 60 Gy | Oral cavity | Dmean < 40 Gy |
| Optic nerves | Dmax ≤ 54 Gy | Submandibular gland | Dmean < 35 Gy |
| Optic nerves_PRV | D1cc ≤ 60 Gy | Cochlea | Dmean < 50 Gy |
| Optic chiasm | Dmax ≤ 54 Gy | Superior constrictor of pharynx | Dmean < 45 Gy |
| Optic chiasm_PRV | D1cc ≤ 60 Gy | Middle constrictor of pharynx | Dmean < 45 Gy |
| Temporal lobe | Dmax ≤ 60 Gy | Inferior constrictor of pharynx | Dmean < 45 Gy |
| Temporal lobe_PRV | D1cc ≤ 65 Gy | Larynx | Dmean < 45 Gy |
| Lens | Dmean**‡** < 8 Gy | Trachea | Dmean < 45 Gy |
| Pituitary | Dmax < 60 Gy | Oesophagus | V35 < 50% |
| Thyroid | Dmean < 35 Gy | Mandible | Dmax < 70 Gy |
| Eyes | Dmean < 35 Gy |  |  |

PRV = planning organ at risk volume. ^*^Maximum point dose to the target volume. † Dose received by 1 cubic centimetre of the target volume. ‡ Mean dose to the target volume. § At least 50% of the gland will receive < 30 Gy (should be achieved in at least one gland).
